# Intrahemispheric White Matter Asymmetries and Interhemispheric Connections Underlying the Lateralization of Language Production and Spatial Attention in Left-Handers

**DOI:** 10.1162/nol_a_00153

**Published:** 2025-01-10

**Authors:** Miaomiao Zhu, Xiao Wang, Xier Zhao, Qing Cai

**Affiliations:** Key Laboratory of Brain Functional Genomics (MOE & STCSM), Shanghai Changning-ECNU Mental Health Center, School of Psychology and Cognitive Science, East China Normal University, Shanghai, China; Institute of Brain Science and Education Innovation, East China Normal University, Shanghai, China; NYU-ECNU Institute of Brain and Cognitive Science, New York University Shanghai, Shanghai, China

**Keywords:** corpus callosum, functional lateralization, language lateralization, language production, spatial attention, superior longitudinal fasciculus

## Abstract

Leftward language production and rightward spatial attention are salient features of functional organization in most humans, but their anatomical basis remains unclear. Interhemispheric connections and intrahemispheric white matter asymmetries have been proposed as important factors underlying functional lateralization. To investigate the role of white matter connectivity in functional lateralization, we first identified 96 left-handers using visual half field naming tasks. They were then divided into atypical and typical functional dominance based on the lateralization of brain activation in a word generation task (for language production) and a landmark task (for spatial attention). Using a novel fixel-based framework, we obtained fiber-specific properties of white matter pathways. Results showed, first, that differences between two language dominance groups occurred in the asymmetry of the superior longitudinal fasciculus-III (SLF-III), whereas differences between two spatial attention dominance groups occurred in the rostrum and rostral body of the corpus callosum. However, the directions of functional lateralization were not associated with the directions of white matter asymmetries. Second, the degree of language lateralization was predicted by SLF-III asymmetry and the rostral body of the corpus callosum, whereas the degree of spatial attention lateralization was predicted by the rostrum of the corpus callosum. Notably, the degree of each functional lateralization was negatively correlated with the anterior and middle callosal connections, supporting the excitatory model of the corpus callosum. The results suggest that language lateralization is shaped by a combined effect of intra- and interhemispheric connections, whereas spatial attention lateralization relies more on interhemispheric connections.

## INTRODUCTION

It is well acknowledged that the functional division of two hemispheres has evolved to facilitate efficient activities crucial for animal survival (Güntürkün & Ocklenburg, 2017; Rogers et al., 2004; Vallortigara & Rogers, 2005). This macroscale feature of brain organization is conserved in human beings. For most people, language is lateralized to the left hemisphere (Knecht et al., 2000; Malik-Moraleda et al., 2022; Mazoyer et al., 2014), while spatial attention is generally lateralized to the right (Heilman & Van den Abell, 1980; Mesulam, 1981). Notably, previous research has observed a shift in the dominance of spatial attention to the left when language dominance is present in the right hemisphere, indicating a complementary lateralization of the two functions (Cai et al., 2013). Reduced functional lateralization has been argued to be a potential factor for various psychiatric and neurodevelopmental disorders (Bishop, 2013). However, despite the clinical significance of functional lateralization, its structural basis remains unclear.

A prevailing hypothesis posits that functional lateralization may arise from the gray matter asymmetry (GMA; Galaburda et al., 1978; Geschwind & Levitsky, 1968; Toga & Thompson, 2003), which has been predominantly investigated in the context of language functions. At the population level, many language-related regions, in particular the planum temporale, inferior frontal gyrus, and Heschl’s gyrus, exhibit leftward asymmetry (Kong et al., 2018; Ocklenburg et al., 2016; Schmitz et al., 2019). However, they are less likely to be directly related to language lateralization, as evidenced by the functional-structural segregation of asymmetries in left-handers with right hemisphere language dominance (Gerrits et al., 2022; Leroy et al., 2015; Tzourio-Mazoyer et al., 2018). Only subtle GMAs in certain areas (i.e., insula, part of planum temporale and the ventral occipitotemporal cortex) were found to be associated with language lateralization (Greve et al., 2013), indicating that GMAs alone were unlikely to be the anatomical drivers of functional lateralization.

Morphological analysis of gray matter in specific regions provides information about localization, but does not capture the distributed nature of functional representation. Conversely, investigating white matter pathways highlights structural connections between isolated brain regions rather than focusing on individual regions. In terms of language production, two dorsal pathways, the left arcuate fasciculus and the superior longitudinal fasciculus-III (SLF-III), which connect the temporal-frontal and parietal-frontal regions, respectively, play a key role in processing phonological, semantic, and articulatory aspects of language (Saur et al., 2008; Yagmurlu et al., 2016). Additionally, the interhemispheric callosal connection not only supports the integration of linguistic features and paralinguistic information of language (Kellmeyer et al., 2019; Levy & Trevarithen, 1977), but also plays a crucial role in syntactic comprehension (Sanders, 1989). Regarding spatial attention, studies have demonstrated that damage to the right SLF II/III can lead to severe deficits in attentional ability, that is, visual neglect (for reviews see Bartolomeo, 2021; Bartolomeo & Seidel Malkinson, 2019; Bartolomeo et al., 2007; Corbetta & Shulman, 2011). Furthermore, the integrity of corpus callosum is critical for effective rehabilitation therapy and recovery from neglect (Lunven et al., 2019; Nyffeler et al., 2019).

Given the critical role of intra- and interhemispheric white matter tracts in language and attention function, it is worth investigating how these two anatomical aspects relate to functional lateralization. For intrahemispheric white matter tracts, the arcuate fasciculus showed leftward asymmetry at the population level (Bain et al., 2019; Catani & Mesulam, 2008; Ocklenburg et al., 2013; Powell et al., 2006). However, its association with language lateralization at the individual level remains unclear (see review in Ocklenburg et al., 2016). Likewise, for spatial attention, the SLF-II/III showed co-lateralization with rightward functional laterality. Specifically, SLF-II asymmetry was found to be linked to spatial biases in the bisection task (Kocsis et al., 2019; Thiebaut de Schotten et al., 2011). However, the relationship between functional and white matter asymmetries for spatial attention has yet to be directly examined. For interhemispheric connections, two opposing models have been proposed regarding the role of the corpus callosum in the establishment of functional lateralization (van der Knaap & van der Ham, 2011). The excitatory model posits that the corpus callosum facilitates information sharing between hemispheres via excitatory callosal connections so as to reduce left–right differences (Gazzaniga, 2000; Ringo et al., 1994). This leads to a reduction in functional lateralization. Evidence supporting this model emerges from studies combining behavioral paradigms or task-based functional magnetic resonance imaging (fMRI) with diffusion tensor imaging (DTI), which revealed a negative correlation between functional lateralization and callosal connectivity (Gootjes et al., 2006; Putnam et al., 2008; Westerhausen & Hugdahl, 2008). Conversely, the inhibitory model proposes that the corpus callosum serves to sustain the independence of two hemispheres, preventing mutual interference through reciprocal inhibition to the contralateral area (Cook, 1984), which results in enhanced lateralization. Support for this model arises from studies of individuals with split-brains or agenesis of the corpus callosum, who showed more bilateral language activation compared to controls (Hinkley et al., 2016; Komaba et al., 1998). This model also obtains support in studies involving healthy individuals that displayed a positive function–structure relationship (Josse et al., 2008).

Overall, the understanding of white matter connectivity underlying functional lateralization is far from clear. One important reason is that most studies have focused primarily on right-handed populations, who predominantly exhibit a typical lateralization pattern (i.e., leftward language and rightward attention dominance). Consequently, those studies were unable to compare and dissociate the anatomical difference between individuals with typical and atypical lateralization. Considering left-handers exhibit a higher prevalence of atypical language lateralization (∼25%) compared to right-handers (∼5%), their inclusion in research can provide unique insights into the functional interactions and underlying anatomical basis of lateralization (Cai & Van der Haegen, 2015; Willems et al., 2014). A study by Cai et al. (2013) examined 28 left-handers with left or right language dominance (LLD or RLD, respectively), and uncovered a complementary pattern of functional lateralization between language production and spatial attention. Notably, a scarcity of anatomical studies has encompassed left-handed individuals. For instance, Gerrits et al. (2022) and Vernooij et al. (2007) enrolled 63 and 13 left-handers, respectively, to explore white matter asymmetries underlying language lateralization. Both studies reported no significant difference in the asymmetry of the arcuate fasciculus between left-handers with typical and atypical lateralization. In contrast, Verhelst et al. (2021) identified a significant difference in the anterior callosal connection linking non-language regions. These studies, however, were constrained by their modeling methods or the stringent statistical corrections resulting from the data-driven methods. Hence, further research focusing on left-handed populations and utilizing advanced modeling techniques is required to fully understand the relationship between white matter structure and functional lateralization.

In the present study, we investigated (a) the contribution of two types of white matter connectivity to the degree of functional lateralization, and (b) the difference in intrahemispheric white matter asymmetries and interhemispheric connectivity between individuals with atypical and typical lateralization for language production and spatial attention. Our participants were selected from a pool of 153 left-handers, based on two reliable visual half field naming (VHF) tasks (Van der Haegen et al., 2011). To determine functional lateralization of language production and spatial attention, the fMRI experiment used the word generation task and the landmark task, respectively. To quantify the white matter connectivity, we implement an advanced fixel-based analysis framework. This technique utilizes constrained spherical deconvolution (CSD; Tournier et al., 2004) to estimate fiber orientation distributions (FODs) within each voxel. Compared to conventional DTI methods, this approach allows a more accurate estimation of multiple fiber population orientations within each voxel, particularly in crossing-fiber areas (Mito et al., 2018; Verhelst et al., 2021). Notably, it facilitates the assignment of the resulting metrics to specific fiber tracts, thus enhancing the biological interpretation (Dell’Acqua & Tournier, 2019; Tournier et al., 2007). Three quantitative measures were computed: the microscopic fiber density (FD), reflecting the total intra-axonal volume along fiber orientation; the macroscopic fibre-bundle cross-section (FC), capturing the volume perpendicular to fibre orientation; and the combined metric, fiber density cross-section (FDC), computed as the product of FD and FC, serving as a comprehensive indicator of the capacity to transmit information (Raffelt et al., 2017).

Based on prior research and theoretical frameworks, we hypothesize that (1) there is a significant association between the direction of intrahemispheric tract asymmetry and the direction of functional lateralization. Specifically, we expect to observe a right lateralization pattern (right > left FDC/FC/FD) in the atypical language lateralization group, contrasting with a left lateralization pattern in the typical group. For spatial attention, an inverse pattern is expected. Furthermore, a significant group effect on hemispheric differences in fiber asymmetry degree as well as on interhemispheric connectivity (FDC/FC/FD) is anticipated. (2) Regarding the role of the corpus callosum in functional lateralization, we predict that if its influence is inhibitory, connectivity (FDC/FD/FC) will be stronger with greater degrees of functional lateralization. Conversely, if the influence is excitatory, the relationship will be reversed. For intrahemispheric tracts, we expect that a stronger degree of laterality will correlate with a greater degree of functional lateralization.

## MATERIALS AND METHODS

The experimental protocol consisted of several sequential steps. First, a group of left-handers participated in the behavioral screening test. Then, a subset of them proceeded to undergo an MRI scanning session. Finally, the statistical analysis was conducted to assess the relationship between intra- and interhemispheric white matter measurements and the lateralization of functional activity in two fMRI tasks.

### Participants

Participants were recruited through social media, posters, and word-of-mouth, targeting undergraduate students and employed college graduates who self-reported as left-handed. All participants were native Chinese speakers with normal or corrected-to-normal vision and had no reported history of brain injury or psychiatric disorders, including epilepsy or tumors. Handedness was assessed using the Chinese version of the Edinburgh Handedness Inventory (Yang et al., 2018). Based on previous research of left-handedness (Frässle et al., 2016; Mazoyer et al., 2016; Wang et al., 2019) and considering the relatively low incidence of left-handedness in the Chinese population (Fernandez-Velasco et al., 2023; Teng et al., 1976), a moderate threshold of −20 was used for determining left-handedness. This study was approved by the Ethical Committee of East China Normal University. Each participant gave informed consent before the experiment.

Following the protocols from previous studies (Cai et al., 2013; Van der Haegen et al., 2011), a total of 153 left-handers (68 males; mean age, 22.5 yr; age range, 18–34 yr) participated in the VHF picture and word naming tasks. These tasks were validated as effective screening tools for the preliminary identification of individuals with atypical language lateralization, offering a cost-effective approach for following fMRI sessions. Participants were selected based on visual field advantage (VFA; calculated by reaction time [RT] differences between the left and right visual field) at a specified threshold (for details, see Behavioral Screening).

Ninety-six participants underwent the subsequent MRI session. All anatomical images of the subjects were thoroughly checked to identify any potential brain diseases. One participant was excluded due to the presence of an unidentified shadow in the temporal lobe. For functional scans, participants with excessive head movements (one for word generation task, two for landmark task) or limited brain activation in regions of interest (ROIs; two for word generation task and one for landmark task) were excluded. For diffusion scans, four participants were excluded due to significant head motion or signal dropout. One participant was excluded because the diffusion-weighted images (DWIs) failed to complete all pipeline steps. A final cohort of 90 subjects (53 females, mean age: 22.3 yr, age range: 18–34 yr) entered the further analysis.

### Stimuli and Procedure

#### Behavioral screening

##### VHF word naming task.

The stimuli consisted of 192 one-character nouns. Half were used as targets and the other half as fillers to form matched noun pairs. Targets and fillers were pairwise controlled for word frequency, age of acquisition, familiarity, concreteness, and number of strokes based on the Single Character Word Database (SCWD; all *Ps* > 0.36; Liu et al., 2007). Each word pair was repeated twice, with the target and the filler displayed in left or right visual field. This led to a total of 192 trials, split across two runs. All words from the first run were presented again in the second run. Two different trial lists were generated, and the order of presentation was counterbalanced among participants.

##### VHF picture naming task.

Based on Van der Haegen et al. (2011), five line drawings of inanimate objects were created: a book (书-Shu), a flower (花-Hua), a bowl (碗-Wan), a boat (船-Chuan), a lamp (灯-Deng). All names were one-character words, and every picture was symmetrical to avoid bias to either VHF. Each picture formed four pairs by combining with the others. In each pairing, a picture was used as a target or a filler, displayed in either the left or right visual field. Thus, all possible stimulus combinations generated 40 trials in total. These 40 trials were repeated four times in a randomized sequence throughout the experiment.

Participants were seated at a distance of ∼60 cm from the monitor. Each stimulus pair was presented symmetrically relative to the center of the screen using E-prime software (Psychology Software Tools, n.d.). Each trial began with a fixation for 500 ms, followed by 200 ms of bilateral targets and fillers with a central arrow. Participants were instructed to name the target picture or word to which the arrow pointed as quickly and accurately as possible. The onset time of each participant’s voice response was recorded using a series response box (SRBox). Pictures were displayed at a visual angle ranging between 1.91° and 10.93°. Words were displayed in 24 point Song font and spanned visual angles of 2.21° and 3.06° from the screen center to their inner edge and outer edge, respectively. These stimuli in the bilateral fields were then masked by randomly oriented lines with matched sizes in the picture naming task and by two ASCII codes 35 (##) in the word naming task. After the mask’s disappearance, an underline replaced the arrow and remained on the screen for 2,600 ms until a response was collected via the voice key. Breaks were interspersed every 16 trials. Prior to the experiment, participants undertook eight practice trials for picture naming and 16 for word naming.

Trials with response errors or unrecorded responses (subjective omission or SRBox voice key failures) were discarded. For each visual field, trials with RTs exceeding 2.5 standard deviations from the mean were excluded. Based on previous studies (Gerrits, De Clercq, et al., 2020; Van der Haegen et al., 2011), we employed a tiered selection strategy based on VFA values to ensure a diverse range of lateralization across both direction and degree within our participant cohort. Initially, individuals with a VFA less than −20 ms (suggestive of strong RLD, *N* = 50) in either the word or picture naming tasks were prioritized for inclusion in fMRI sessions. Subsequently, participants with a VFA ranging from −20 ms to 10 ms in either task (indicative of weak language lateralization, *N* = 39) were considered. Finally, those with a VFA greater than 10 ms (indicative of left language dominance, *N* = 7) were included.

#### fMRI tasks

##### Word generation task.

This task was used to determine language lateralization, which was selected due to its capacity to capture the lateralization with strong robustness (Bradshaw et al., 2017; Cai et al., 2013; Wang et al., 2019). It consisted of 10 Pinyin blocks, 10 baseline blocks, and 20 additional rest blocks, each lasting 15 s. Pinyin and baseline blocks were presented alternately, with each followed by a rest block. Participants were instructed to covertly generate as many one-character words as possible that began with the letter presented at the screen’s center (Pinyin block) or to repeat the non-lexical sound “bou” when the symbol ˆ was presented (baseline block).

##### Landmark task.

This task was used to determine the lateralization of spatial attention. This task comprised 6 bisection blocks, 6 touch blocks, and 6 rest blocks, each lasting 21.6 s. Bisection and touch blocks were displayed alternately, with every two blocks followed by a rest block. Each block began with a 4 s instruction indicating the type of the task, followed by 12 trials. In each trial, a spatial stimulus appeared for 1.6 s, followed by a 200 ms fixation. In the bisection block, a 15 cm horizontal line and a short vertical line were presented simultaneously. The position of the vertical line relative to the horizontal line was at the middle (50% of trials) or 2.5%, 5%, and 7.5% left or right deviation (50% of trials) of the length. Participants were asked to judge whether or not the vertical line exactly bisected the horizontal line by pressing “1” or “2” with their left index finger. In the touch block, identical stimuli were used, but in half of the trials, the vertical line did not touch the horizontal line. Participants were required to decide whether the two lines touched or not by pressing “1” or “2.”

#### MRI acquisition

Multimodal MRI data were collected on a 3T Siemens Prisma scanner with a 20-channel head coil. T1-weighted anatomical images were acquired with an MPRAGE sequence (repetition time [TR] = 2,300 ms, echo time [TE] = 2.32 ms, voxel size = 0.9 * 0.9 * 0.9 mm^3^, field of view [FOV] = 240 mm, matrix size = 256 * 256, flip angle [FA] = 9°). T2*-weighted functional data were acquired with an echo planar imaging (EPI) sequence (TR = 2,450 ms, TE = 30 ms, voxel size = 3 * 3 * 3 mm^3^, FOV = 192 mm, matrix size = 64 * 64, FA = 81°, 40 axial slices).

High angular resolution diffusion imaging (HARDI) data were acquired using single-shot EPI sequence (b = 1,000/2,000 s/mm^3^, each with 64 directions, one pair of b = 0 volumes with reversed phase encoding directions, TR = 4,200 ms, TE = 70 ms, voxel size = 3 * 3 * 3 mm^3^, FOV = 192 mm, matrix size = 64 * 64, FA = 81°, 40 slices).

#### fMRI data analysis

Functional images were pre-processed using the SPM12 software in MATLAB. The steps included slice-timing, spatial realignment, co-registration, normalization, and smoothing with a 6 mm FWHM Gaussian kernel. To correct for motion artifacts, the ART toolbox (Nicolae et al., 2018) was used to identify the volumes with intensity values exceeding 4 *SD* or with motion greater than 2 mm. The identified outliers were modeled as regressors, together with the motion parameters obtained from the realignment step. Datasets with more than 10% outliers were excluded. In the first level analysis, the experimental design was convolved with a canonical hemodynamic response function to generate task-related regressors for the general linear model.

Lateralization index (LI) of language lateralization was computed for each participant based on the brain activation during Pinyin versus baseline condition in the word generation task. The pars triangularis and pars opercularis (BA 44 and BA 45) were selected as the ROIs. These regions play critical roles in the phonological, semantic, and articulatory processes of language functions and consistently exhibit salient activation during language-related tasks (Heim et al., 2008; Sahin et al., 2009). The LI of spatial attention was derived from the activation during bisection versus touch condition in the landmark task, with the inferior parietal lobe and the superior parietal lobe as ROIs. To generate symmetric masks of the ROIs, the original regions in the automated anatomical labeling atlas were superimposed with the corresponding left–right flipped ones. Moreover, a small volume correction (*p* < 0.05) was applied to each ROI, excluding individuals with fewer than 10 surviving voxels to reduce the effect of noise on LI calculation. LIs were calculated using the LI toolbox (Wilke & Lidzba, 2007; Wilke & Schmithorst, 2006). Briefly, a bootstrap method was used to generate 100 samples in bilateral ROIs at multiple *t*-value thresholds, resulting in 10,000 LI (left − right / (left + right)) combinations. Only the central 50% of the data points were extracted to obtain a trimmed mean. Finally, a weighted mean LI was then computed for each participant, giving more weights to higher thresholds.

Based on the LI sign, participants were divided into two groups. Those with negative LI were defined as the right lateralization group and those with positive LI were classified as the left lateralization group. The degree of lateralization was assessed using the absolute value of the LI. For language production, 27 subjects were divided into right dominance group, 60 subjects were divided into left dominance group. For spatial attention, 51 subjects were classified as right dominance group, 35 subjects were classified as left dominance group. Note that the lateralization of the two functions is not always perfectly complementary. An individual might be typically lateralized for one function, such as language production, but may fall into the atypical group for another function, such as spatial attention.

#### Diffusion-weighted images preprocessing and fixel-based metrics

DWIs were preprocessed according to the fixel-based analysis pipeline (Dhollander et al., 2021; Raffelt et al., 2017). This consisted of denoising, Gibbs ringing removal, correction for head motion, eddy current, and susceptibility-induced EPI distortion, as well as bias field correction, upsampling, and generation of brain masks. Quality control was performed using eddy QC tools (Bastiani et al., 2019), excluding datasets with excessive head movement (>6% of volumes with over 1.5 mm estimated displacement) or signal dropout (>6% of volumes with over 8% dropout-slices).

By using a robust unsupervised method (Dhollander et al., 2019), response functions for three tissues (white matter, grey matter and cerebrospinal fluid) were obtained for each subject. These response functions were averaged across subjects to establish a unique set of group-level response functions. Subsequently, they were used to calculate FODs using multishell–multitissue constrained spherical deconvolution (Dhollander & Connelly, 2016). After global intensity normalization of the FODs, a study-specific, unbiased symmetric FOD template was created using images from 30 subjects (15 individuals with left hemispheric dominance for language and right hemispheric dominance for spatial attention, and another 15 individuals with right hemispheric dominance for language and left hemispheric dominance for spatial attention) and their left-to-right flipped counterparts via an iterative registration method (Raffelt et al., 2011). Next, each participant’s FOD images were then warped to the template space and segmented to generate discrete fixels. The fixels for each participant were then re-oriented and assigned to the corresponding fixels in the template (Raffelt et al., 2017). Lastly, fixel-based maps (FD, logFC, and FDC) were calculated for each subject and transformed into population space using subject-to-group FOD template transformation.

#### Tractography

Considering that the subregions of the corpus callosum and their corresponding projected brain areas may play distinct roles in various cognitive functions, we segmented the corpus callosum into seven subdivisions based on the definition of Witelson (1989). This helped us further investigate the relationship between functional lateralization and interhemispheric connections. Corpus callosum-1 (Rostrum) connects bilateral orbitofrontal areas, corpus callosum-2 (Genu) connects bilateral prefrontal areas, corpus callosum-3 (Rostral body) connects bilateral premotor area, corpus callosum-4 (anterior midbody) connects bilateral motor areas, corpus callosum-5 (posterior midbody) connects bilateral postcentral areas, corpus callosum-6 (Isthmus) connects bilateral superior temporal lobule, posterior parietal lobule, and isthmus. Corpus callosum-7 (Splenium) connects bilateral occipital and inferior temporal lobules. For intrahemispheric fiber pathways, two language pathways were reconstructed. The arcuate fasciculus connects the temporal lobe with the inferior frontal gyrus, middle frontal gyrus, and precentral gyrus. The SLF-III connects the supramarginal gyrus and the inferior frontal gyrus. Additionally, the SLF-II, which connects the inferior parietal lobe and dorsolateral frontal region, was also reconstructed as the tracts of interest (TOI) for spatial attention along with the SLF-III.

All tracts were reconstructed using TractSeg (Wasserthal et al., 2018), a deep-learning based segmentation approach, which reaches a balance between the accuracy of user-defined delineation and the reliability of atlas-based tractography. Based on the group-specific FOD template, MRtrix CSD peaks were created and used as input to TractSeg, which generated three segmentations (tracts bundle, end-regions, and start-regions) and tract orientation maps for probabilistic tracking. Each TOI was produced with 10,000 streamlines, from which tract density images (TDIs) were derived. The TDIs were then binarized to obtain a fixel mask for each tract. Finally, average FDC, logFC, and FD were computed across fixels within each tract for each participant. The intrahemispheric white matter asymmetry index was assessed using the formula: (left − right) / (left + right) (for logFC only L − R). The positive asymmetry index indicates a leftward asymmetry in the white matter tract, whereas the negative asymmetry index indicates a rightward asymmetry.

To examine the validity of two VHF naming tasks in identifying individuals with varing brain lateralization, Spearman’s correlation analysis was tested between behavioral index and LI of brain activation.

We conducted a comprehensive investigation into the relationship between the corpus callosum, intrahemispheric white matter asymmetries, and functional lateralization, considering both the degree and the direction of the lateralization. First, Spearman’s rank correlation tests were performed to examine the association between functional lateralization and tract symmetry for each TOI and each function. Second, to examine the potential link between the direction of functional lateralization and the direction of the white matter symmetry, chi-square tests were performed for each function. Third, the nonparametric Wilcoxon test was conducted to examine the differences of the white matter asymmetries between individuals with typical and atypical lateralization directions. Additionally, a two-way analysis of variance (ANOVA) was conducted with hemisphere as the within-subjects variable and lateralization group as the between-subjects variable. The *p* values obtained were subsequently adjusted using the Benjamini-Hochberg false discovery rate (FDR) correction method, separately for inter- and intrahemispheric connectivity within each function, with a significance threshold of 0.05. Furthermore, to investigate the combined effect of the degree of intrahemispheric tracts asymmetries and callosal connectivity on the degree of functional lateralization, Bayesian regression models were developed for the three fixel-based metrics. These models were particularly chosen to mitigate overfitting concerns given the relatively small sample size (*N* = 87) and collinearity between independent variables (van de Schoot & Depaoli, 2014). Four independent Markov chain Monte Carlo chains were run for each model. The chains consisted of 50,000 iterations, with the first half removed as the burn-in phase. Model convergence was assessed using R-hat values, where a value of 1.10 or less indicates convergence. Trace plots were also visually inspected. Default noninformative priors from the brms package (Bürkner, 2017) were utilized. For each predictor, median of estimates and 95% credibility intervals (CI) were derived from the posterior distributions. The probability of direction (*pd*) was included to quantify the likelihood of the effect being in a certain direction. The *pd* is strongly correlated with the frequentist *p* value (a *pd* of 95%, 97.5%, 99.5%, and 99.95% correspond to a two-sided *p* value of 0.1, 0.05, 0.01, and 0.001, respectively). The percentage in the region of practical equivalence (ROPE) reported as a continuous index of significance, with values below 1% are considered significant, allowing to confidently reject the null hypothesis. In these models, the absolute functional LI served as the dependent variable, with the absolute asymmetry index of the intrahemispheric pathways and interhemispheric connectivity as independent variables. Furthermore, additional independent variables were also included to examine the potential interaction effects of the directionality consistency of function–structure asymmetry and the degree of tracts asymmetry. Gender and age were also incorporated in the models. All statistical analyses and visualizations were performed using R packages (correlation, brms and ggplot2; R Core Team, 2023; for packages see also, respectively, Bürkner, 2017; Makowski et al., 2020; Wickham et al., 2024).

## RESULTS

### Association Between Behavioral Laterality and Functional Lateralization

**Figure 1.**
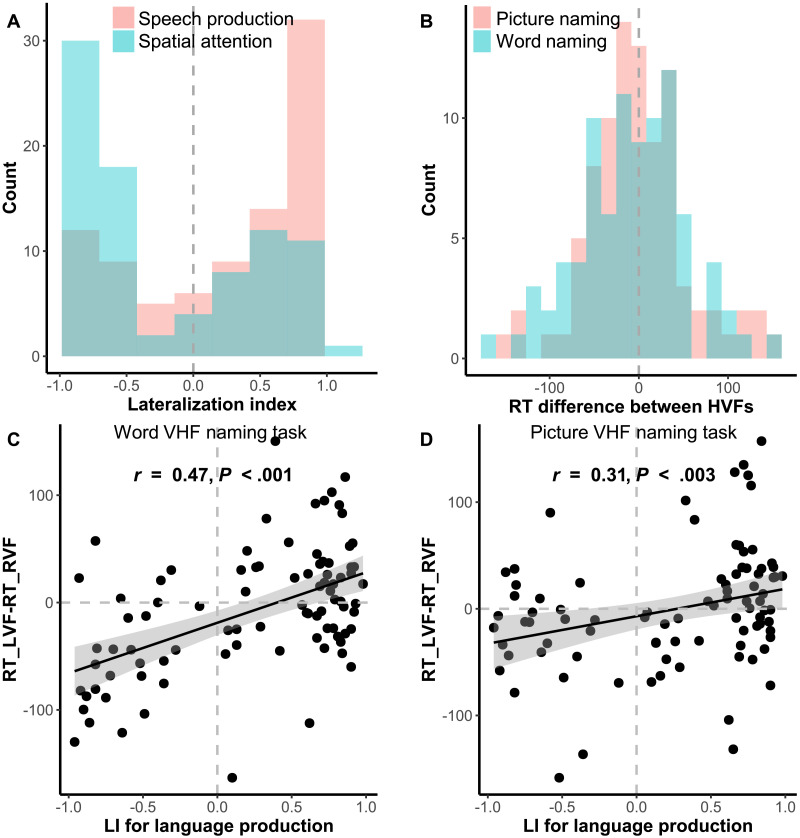
Correlations between functional lateralization index (LI) in language production and reaction time (RT) differences between two visual half fields (VHFs) in word and picture naming tasks. **A.** Both the LI for language production and spatial attention present a non-normally distribution. **B.** RT differences in the word naming task displays a normal distribution, whereas those in the picture naming task displays a non-normal distribution. **C–D.** The LI of brain activation based on Broca’s area is positively correlated with RT differences in two naming tasks. Despite the high false alarm rate, the behavioral screening identified atypical lateralization individuals with a high hit rate and enough individuals with weak laterality. RT_LVF-RT_RVF indicates reaction time differences between the left and right VHF; LI indicates language dominance: below 0 for right, above 0 for left. The closer to −1 or 1, the stronger the dominance.

To evaluate the utility of the VHF picture and word naming tasks in measuring language dominance in Chinese, we conducted a Spearman’s rank correlation analysis between the RT difference of the two VHF naming tasks (Picture: *M* = −0.63, *SD* = 56.49; Word: *M* = −7.78, *SD* = 58.72) and the functional LI (*M* = 0.25, *SD* = 0.64) in the word generation task. The normality of both the behavioral performance and the LI was assessed using the Shapiro-Wilk distribution test. As shown in Figure 1A–B, both the LI of language production and spatial attention were non-normally distributed with right and left skewness (*W* = 0.85, *p* < 0.001; *W* = 0.85, *p* < 0.001, respectively). The RT difference between two VHFs in the word VHF task showed a normal distribution (*W* = 0.99, *p* > 0.05), whereas the RT difference in the picture VHF task showed a non-normal distribution (*W* = 0.97, *p* = 0.04). Furthermore, significant correlations were observed between the LI derived from the brain activation of Broca’s area in the fMRI task and the RT differences between two VHFs in the naming tasks (Picture: *r* = 0.31, *p* < 0.003; Word: *r* = 0.47, *p* < 0.001; Figure 1C–D). Despite the high false alarm rate (i.e., 43% for word naming, 48% for picture naming) in our participants, the behavioral screening identified individuals with atypical brain lateralization with a high hit rate (i.e., 79% for word naming, 74% for picture naming) and enough individuals with weak laterality.

We also conducted a correlation analysis to explore the relationship between handedness laterality, behavioral laterality, and functional lateralization. No significant correlation was found between handedness and the other two variables (all *p* values > 0.05; see Supplementary Figure 1 in the Supporting Information, available at https://doi.org/10.1162/nol_a_00153). In addition, we found no statistically significant differences in handedness laterality quotient between the language groups (right dominance group: *M* = −0.5, *SD* = 0.19; left dominance group: *M* = −0.55, *SD* = 0.24; *p* = 0.27) and the spatial attention groups (right dominance group: *M* = −0.48, *SD* = 0.19; left dominance group: *M* = −0.53, *SD* = 0.25; *p* = 0.14).

### Comparison of Intrahemispheric Pathway Asymmetry and Callosal Connectivity Between Typical and Atypical Lateralization Groups

**Figure 2.**
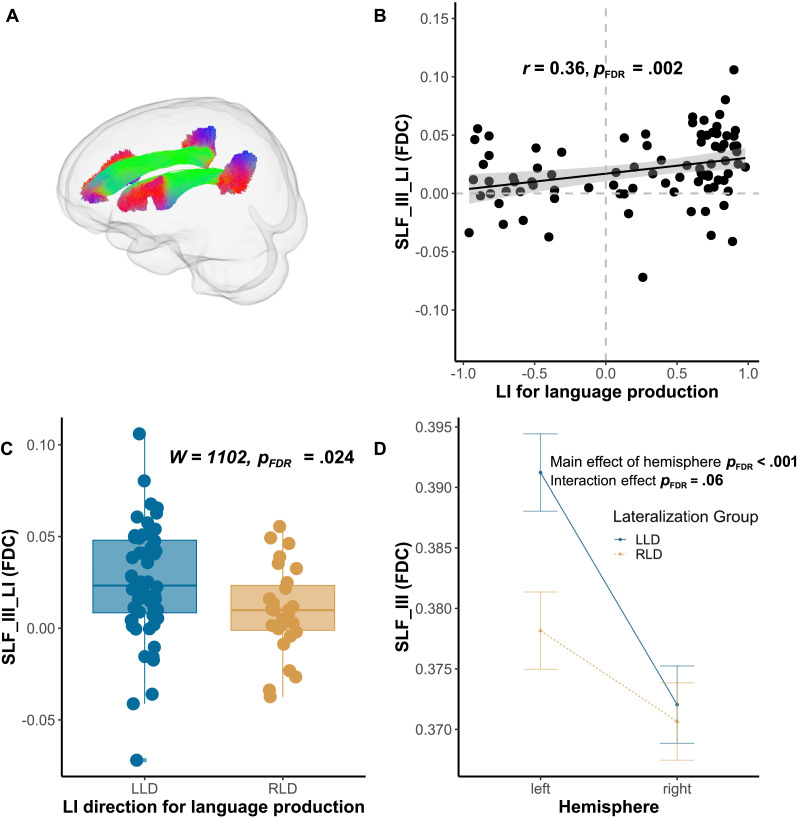
The relationship between functional lateralization in language production and the asymmetry of superior longitudinal fasciculus-III (SLF-III). **A.** The SLF reconstructed used TractSeg. **B.** The lateralization of language production is positively associated with the asymmetry of SLF-III. **C.** Individuals with right language dominance (RLD) showed a less leftward SLF-III in fiber density cross-section (FDC) compared to those with left language dominance (LLD). **D.** The interaction plot of the Hemisphere * Lateralization Group ANOVA. A negative asymmetry index in the plots signifies right lateralization.

For language production, as shown in Table 1 and Figure 2B, the language LI is positively correlated with SLF_III FDC asymmetry (*r* = 0.36, *p*_FDR_ = 0.002) and logFC asymmetry (*r* = 0.36, *p*_FDR_ = 0.002). No correlation was revealed between arcuate fasciculus asymmetry and language LI in either metric (*p*s > 0.05). Furthermore, chi-square tests of independence showed that there was no significant association between functional lateralization direction and the white matter asymmetry direction (*X*^2^ (2, *N* = 87) < 6, *p* > 0.05), as detailed in Supplementary Table 2 and Table 3 in the Supporting Information. Further, the group differences were found only for intrahemispheric pathway asymmetry. As shown in Figure 2C and Table 2, the atypical right language lateralization group (*M* = 0.010, *SD* = 0.02) displayed a significantly lower asymmetry index of the SLF-III FDC compared to the typical left lateralization group (*M* = 0.025, *SD* = 0.03; *W* = 1102, *p*_FDR_ = 0.024). Similar group differences were also observed in SLF-III logFC (*W* = 1069, *p*_FDR_ = 0.027). No such group difference was found for the metrics of the remaining TOIs. In the ANOVA results presented in Table 3 and Figure 2D, significant hemisphere effects were observed for both the FDC and FD of SLF_III, with *F*(1, 85) = 29.49, *p*_FDR_ < 0.001 and *F*(1, 85) = 58.17, *p*_FDR_ < 0.001, respectively. Additionally, there was a marginally significant Hemisphere x Group interaction effect for the SLF_III FDC. Specifically, the left language dominance group (*M* = 0.39, *SD* = 0.04) exhibited larger left SLF_III relative to RLD group (*M* = 0.38, *SD* = 0.04), whereas no difference was found in the right SLF_III between left language dominance group (*M* = 0.37, *SD* = 0.03) and right dominance group (*M* = 0.37, *SD* = 0.04).

**Table 1.** Results of Spearman’s rank correlations between intrahemispheric tracts asymmetry and functional laterality for language production

Variables	*r*	95% CI		*p*	*p*_adj
		LL	UL		
FDC					
SLF_III_AI	0.36	0.15	0.53	<0.001	**0.002**
AF_AI	0.06	−0.15	0.28	0.554	0.554
FD					
SLF_III_AI	0.12	−0.10	0.33	0.252	0.302
AF_AI	−0.17	−0.37	0.05	0.117	0.176
logFC					
SLF_III_AI	0.36	0.16	0.54	<0.001	**0.002**
AF_AI	0.24	0.02	0.43	0.028	0.056

*Note*. FDC = fiber density and cross section, FD = fiber density, FC = fiber cross section, SLF_III_AI = superior longitudinal fasciculus III asymmetry index, AF_AI = arcuate fasciculus asymmetry index. *r* = Spearman correlation coefficient, CI = confidence interval, LL = lower limit, UL = upper limit, *p* = uncorrected *p* value, *p*_adj = adjusted *p* value using the Benjamini-Hochberg procedure for multiple testing.

**Table 2.** Results of between group comparisons of intrahemispheric tracts asymmetry index for language production

Variables	LLD (*N* = 60)		RLD (*N* = 27)		*W*	*p*	*p*_adj
	*M*	*SD*	*M*	*SD*			
FDC							
SLF_III_AI	0.025	0.03	0.010	0.02	1,102	0.004	**0.024**
AF_AI	−0.003	0.02	−0.004	0.02	801	0.535	0.642
FD							
SLF_III_AI	0.019	0.02	0.016	0.02	891	0.230	0.345
AF_AI	−0.005	0.01	0.001	0.01	591	0.978	0.978
logFC							
SLF_III_AI	0.017	0.04	−0.007	0.04	1,069	0.009	**0.027**
AF_AI	0.004	0.03	−0.008	0.03	1,005	0.037	0.074

*Note*. FDC = fiber density and cross section, FD = fiber density, FC = fiber cross section, SLF_III_AI = superior longitudinal fasciculus III asymmetry index, AF_AI = arcuate fasciculus asymmetry index, RLD = right language dominant group, LLD = left language dominant group, *W* = Mann–Whitney statistic, *p* = uncorrected *p* value, *p*_adj = adjusted *p* value using the Benjamini-Hochberg procedure for multiple testing.

**Table 3.** The results of Hemisphere * Group ANOVA analysis for language production

Measures	Effects	*F*(1, 85)	*p*	*p*_adj	*η* ^2^
FDC					
SLF_III	Group	0.77	0.384	0.788	0.008
	Hemisphere	29.49	**<0.001**	**<0.001**	0.027
	Hemisphere * Group	5.64	0.020	**0.060**	0.005
AF	Group	0.073	0.788	0.788	0.001
	Hemisphere	2.350	0.129	0.215	0.002
	Hemisphere * Group	0.005	0.944	0.944	0.000
FD					
SLF_III	Group	1.05	0.308	0.788	0.010
	Hemisphere	58.17	**<0.001**	**<0.001**	0.107
	Hemisphere * Group	0.34	0.563	0.672	0.001
AF	Group	0.179	0.674	0.788	0.002
	Hemisphere	2.187	0.143	0.215	0.003
	Hemisphere * Group	4.649	**0.034**	**0.068**	0.006
logFC					
SLF_III	Group	0.69	0.410	0.788	0.007
	Hemisphere	1.07	0.304	0.360	0.001
	Hemisphere * Group	5.78	**0.018**	**0.060**	0.005
AF	Group	0.086	0.770	0.788	0.001
	Hemisphere	0.442	0.508	0.508	0.000
	Hemisphere * Group	3.098	0.082	0.123	0.002

*Note*. FDC = fiber density and cross section, FD = fiber density, FC = fiber cross section, SLF_III = superior longitudinal fasciculus III, AF = arcuate fasciculus, *p* = uncorrected *p* value, *p_*adj = adjusted *p*-value using the Benjamini-Hochberg procedure for multiple testing.

**Figure 3.**
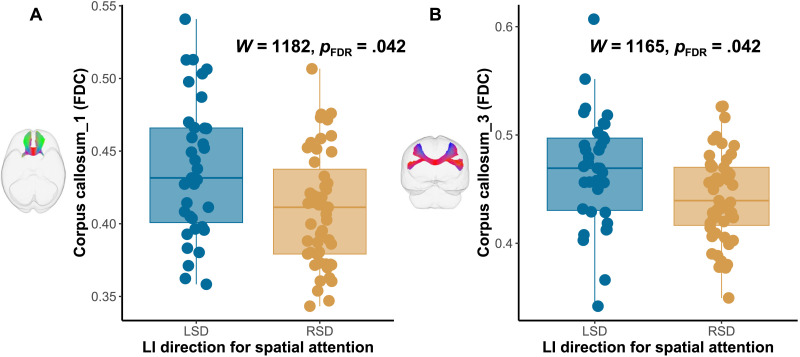
Difference in the asymmetry of SLF_III between two groups of spatial attention lateralization. **A.** Rostrum. **B.** Rostral body. The left spatial attention dominance (LSD) group showed a higher FDC of the rostrum and rostral body compared to the right spatial attentional dominance (RSD) group.

**Figure 4.**
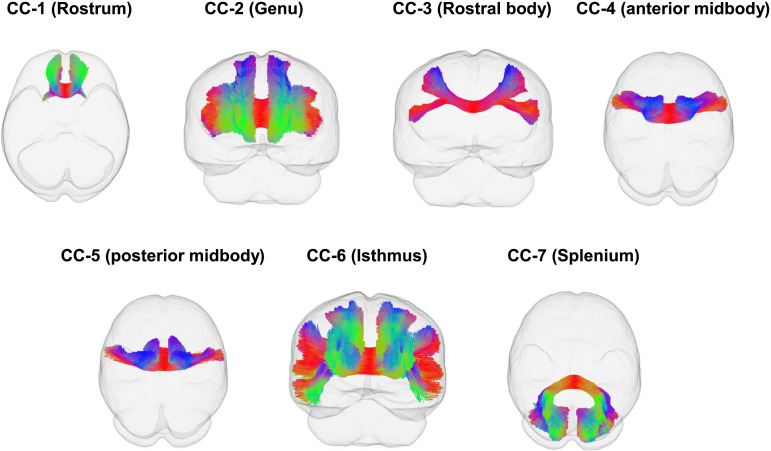
The subdivisions of the corpus callosum (CC) reconstructed using TractSeg.

For spatial attention, there were negative correlations observed between SLF_III FDC (*r* = −0.29, *p*_FDR_ = 0.024) asymmetry and SLF_III logFC asymmetry (*r* = −0.33, *p*_FDR_ = 0.012). However, when controlling for the language lateralization, these correlations ceased to be significant, as showed in Supplementary Table 1 (all *p*_FDR_ > 0.05). Additionally, the chi-square tests of independence revealed no significant association between the direction of functional lateralization and the direction of white matter asymmetry (*X*^2^ (2, *N* = 86) < 6.3, *p* > 0.05, see Supplementary Table 4 and Table 5 in the Supporting Information). Furthermore, group differences were found only for interhemispheric callosal connectivity. As shown in Figure 3 and Table 4, there were larger FDC for rostrum (*W* = 1,182, *p*_FDR_ = 0.042) and rostral body (*W* = 1,165, *p*_FDR_ = 0.042) for the left visuospatial lateralization group (*M* = 0.44, *SD* = 0.05; *M* = 0.47, *SD* = 0.05) compared to the right lateralization group (*M* = 0.41, *SD* = 0.04; *M* = 0.44, *SD* = 0.04). Similarly, there were larger logFC for rostrum (*W* = 1,212, *p*_FDR_ = 0.042) and rostral body (*W* = 1,177, *p*_FDR_ = 0.042) for the left lateralization group (*M* = 0.08, *SD* = 0.08; *M* = 0.06, *SD* = 0.10) compared to the right group (*M* = 0.03, *SD* = 0.07; *M* = 0.02, *SD* = 0.07). No such group difference was found for the remaining parts of the corpus callosum. All subdivisions of the corpus callosum reconstructed were displayed in Figure 4.

**Table 4.** Results of the group comparisons of the connectivity of the corpus callosum subdivisions for spatial attention

Measures	LLD (*N* = 35)		RLD (*N* = 51)		*W*	*p*	*p*_adj
	*M*	*SD*	*M*	*SD*			
FDC							
CC_1	0.44	0.05	0.41	0.04	1,182	0.006	**0.042**
CC_2	0.42	0.04	0.41	0.04	1,136	0.016	0.056
CC_3	0.47	0.05	0.44	0.04	1,165	0.008	**0.042**
CC_4	0.51	0.04	0.50	0.04	1,051	0.082	0.172
CC_5	0.47	0.03	0.46	0.04	984	0.212	0.297
CC_6	0.46	0.04	0.44	0.04	1,010	0.152	0.266
CC_7	0.46	0.06	0.45	0.04	995	0.185	0.278
FD							
CC_1	0.40	0.02	0.40	0.02	1,033	0.109	0.208
CC_2	0.40	0.01	0.40	0.02	928	0.379	0.468
CC_3	0.44	0.02	0.43	0.03	973	0.241	0.316
CC_4	0.49	0.02	0.49	0.02	919	0.410	0.478
CC_5	0.46	0.01	0.46	0.02	792	0.813	0.854
CC_6	0.42	0.02	0.42	0.02	758	0.882	0.882
CC_7	0.41	0.02	0.41	0.02	821	0.737	0.815
logFC							
CC_1	0.08	0.08	0.03	0.07	1,212	0.003	**0.042**
CC_2	0.08	0.08	0.04	0.07	1,139	0.015	0.056
CC_3	0.06	0.10	0.02	0.07	1,177	0.006	**0.042**
CC_4	0.05	0.06	0.02	0.06	1,088	0.043	0.113
CC_5	0.03	0.06	0.01	0.06	1,081	0.049	0.114
CC_6	0.07	0.07	0.04	0.06	1,097	0.037	0.111
CC_7	0.13	0.09	0.12	0.06	996	0.183	0.278

*Note*. FDC = fiber density and cross section, FD = fiber density, FC = fiber cross section, CC_1 to CC_7 = subdivisions of the corpus callosum, RLD = right language dominant group, LLD = left language dominant group, *W* = Mann–Whitney statistic, *p* = uncorrected *p* value, *p*_adj = adjusted *p* value using the Benjamini-Hochberg procedure for multiple testing.

### Relationship Between the Degree of Functional Lateralization, the Degree of Intrahemispheric Pathway Asymmetry, and Callosal Connectivity

To investigate the relationship between the degree of intrahemispheric tracts asymmetry, callosal connectivity, and the degree of functional lateralization, three Bayesian regression models (FD, FC, and FDC) were built for each function. All models indicated good convergence with all R-hat values less than 1.1. For language production, the FDC model is presented in Table 5, which recognized several meaningful predictors. The lateralization degree of SLF_III has a significant positive effect with a probability of 97.85% (Median = 5.72, 95% CI [0.18, 11.24], <1% in ROPE). Conversely, the CC_3 connectivity showed a negative effect with a probability of 97.54 % (Median = −2.45, 95% CI [−4.89, −0.01], 0.07% in ROPE). The FD model in Table 6 showed the significant negative effect of CC_3 connectivity (Median = −5.84, 95% CI [−10.11, −1.57], *pd* = 99.59%, <1% in ROPE). However, the logFC model did not show any significant predictors (see in Supplementary Table 8). Regarding spatial attention, as shown in Table 7 and Table 8, CC_1 connectivity displayed a significant negative effect in logFC model (Median = −1.56, 95% CI [−2.98, −0.15], *pd* = 98.41%, <1% in ROPE) and a marginally negative significant effect of CC_4 connectivity in FD model (Median = −5.46, 95% CI [−11.33, 0.39], *pd* = 96.61%, 0.12% in ROPE) on the degree of functional lateralization. However, the FDC model did not yield any significant predictors (see in Supplementary Table 9).

**Table 5.** Results of Bayesian regression analysis for white matter variables (FDC) predicting the degree of functional lateralization for language production

Parameters	Median	95% CI		*pd*	% in ROPE
		LL	UL		
(Intercept)	0.33	−0.68	1.34	74.26%	3.26%
Gender	−0.01	−0.13	0.11	56.11%	32.30%
Age	−0.01	−0.03	0.01	70.67%	99.54%
FDC_AF_LIa	−4.40	−11.89	3.02	88.01%	0.30%
FDC_SLF_III_LIa	5.72	0.18	11.24	**97.85%**	<0.01%
FDC_CC1	−0.89	−3.16	1.38	77.91%	1.33%
FDC_CC2	3.17	−1.42	7.76	91.43%	0.37%
FDC_CC3	−2.45	−4.89	−0.01	**97.54%**	0.07%
FDC_CC4	1.02	−2.02	4.06	74.68%	1.11%
FDC_CC5	−1.73	−5.36	1.91	82.62%	0.70%
FDC_CC6	2.30	−0.97	5.57	91.75%	0.48%
FDC_CC7	−0.53	−2.71	1.65	68.56%	1.68%
FDC_AF_LIa:FDC_AFLI_direc	−0.32	−9.63	9.10	52.63%	0.47%
FDC_SLF_III_LIa:FDC_SLFIII_direc	−2.84	−9.24	3.57	80.80%	0.43%

*Note*. Median is median estimate of posterior distributions. Probability of direction (*pd*) quantifies the effect’s directional likelihood, correlating with *p* values (*pd* of 95% ≈ *p* < 0.1; 97.5% ≈ *p* < 0.05; 99.5% ≈ *p* < 0.01; 99.95% ≈ *p* < 0.001). % in region of practical equivalence (ROPE) is shown, with <1% considered significant for rejecting the null hypothesis. FDC_AF_LIa indicates the absolute of AF FDC asymmetry; FDC_SLF_III_LIa indicates the absolute of SLF_III FDC asymmetry; FDC_AF_LIa:FDC_AFLI_direc indicates the interaction term between the directionality consistency of language function-AF asymmetry and the AF asymmetry degree; and FDC_SLF_III_LIa:FDC_SLFIII_direc indicates the interaction term between the directionality consistency of language function-SLF-III asymmetry and the SLF-III asymmetry degree. The naming convention used for variables is consistently applied across all regression tables. FDC = fiber density cross-section, CI = credibility interval, LL = lower limit, UL = upper limit, AF = arcuate fasciculus, LI = lateralization index, SLF-III = superior longitudinal fasciculus III, CC_1 to CC_7 = subdivisions of the corpus callosum.

**Table 6.** Results of Bayesian regression analysis for white matter variables (FD) predicting the degree of functional lateralization for language production

Parameters	Median	95% CI		*pd*	% in ROPE
		LL	UL		
(Intercept)	0.21	−1.6	2.05	59.50%	2.24%
Gender	0.03	−0.09	0.16	71.27%	28.91%
Age	−0.01	−0.02	0.02	61.29%	100.00%
FD_AF_LIa	4.08	−4.67	12.88	82.20%	0.30%
FD_SLF_III_LIa	3.66	−2.33	9.66	88.57%	0.32%
FD_CC1	1.04	−4.19	6.35	65.47%	0.76%
FD_CC2	−0.46	−10.88	9.85	53.45%	0.40%
FD_CC3	−5.84	−10.11	−1.57	**99.59%**	<0.01%
FD_CC4	−0.25	−6.34	5.9	53.15%	0.63%
FD_CC5	0.32	−5.64	6.27	54.28%	0.71%
FD_CC6	4.58	−2.72	11.88	89.39%	0.26%
FD_CC7	1.69	−2.83	6.26	77.12%	0.71%
FD_AFLI_direc	0.11	−0.1	0.31	84.49%	11.98%
FD_SLFIII_direc	−0.01	−0.21	0.19	55.36%	20.34%
FD_AF_LIa:FD_AFLI_direc	−6.92	−16.47	2.61	92.40%	0.13%
FD_SLF_III_LIa:FD_SLFIII_direc	−0.13	−7.59	7.39	51.28%	0.55%

**Table 7.** Results of Bayesian regression analysis for white matter variables (FD) predicting the degree of functional lateralization for spatial attention

Parameters	Median	95% CI		*pd*	% in ROPE
		LL	UL		
(Intercept)	3.41	1.7	5.12	100.00%	<0.01%
Gender	−0.09	−0.22	0.03	92.38%	11.89%
Age	<0.01	−0.02	0.02	64.71%	100.00%
FD_SLF_II_LIa	1.14	−3.09	5.38	70.39%	0.92%
FD_CC1	0.53	−4.44	5.5	58.39%	0.77%
FD_CC2	−1.99	−11.51	7.58	65.85%	0.43%
FD_CC3	1.04	−3.04	5.06	69.29%	0.83%
FD_CC4	−5.46	−11.33	0.39	**96.61%**	0.12%
FD_CC5	0.1	−5.6	5.82	51.33%	0.72%
FD_CC6	−1.18	−7.79	5.46	63.85%	0.59%
FD_CC7	1.13	−3.2	5.5	69.86%	0.79%
FD_SLF_III_LIa	1.54	−2.38	5.47	78.43%	0.78%
FD_SLF_II_LIa:FD_SLF_IILI_direc	−3.59	−8.6	1.43	92.14%	0.32%
FD_SLF_III_LIa:FD_SLFIII_direc	1.99	−1.29	5.27	88.54%	0.63%

**Table 8.** Results of Bayesian regression analysis for white matter variables (logFC) predicting the degree of functional lateralization for spatial attention

Parameters	Median	95% CI		*pd*	% in ROPE
		LL	UL		
(Intercept)	0.61	0.11	1.11	99.13%	<0.01%
Gender	<0.01	−0.13	0.14	52.26%	30.02%
Age	<−0.01	−0.02	0.02	56.90%	100.00%
logFC_SLF_II_LIa	1.7	−0.67	4.09	92.11%	0.64%
logFC_CC1	−1.56	−2.98	−0.15	**98.41%**	<0.01%
logFC_CC2	1.78	−0.47	4.00	94.01%	0.55%
logFC_CC3	−0.13	−1.56	1.30	57.14%	2.90%
logFC_CC4	−0.95	−3.16	1.26	80.35%	1.32%
logFC_CC5	0.35	−1.99	2.70	61.75%	1.62%
logFC_CC6	−0.6	−2.35	1.16	74.97%	1.86%
logFC_CC7	0.22	−0.95	1.40	64.62%	3.29%
logFC_SLF_III_LIa	1.12	−1.14	3.39	83.76%	1.19%
logFC_SLF_II_LIa:logFC_SLF_IILI_direc	0.06	−2.21	2.31	52.03%	1.82%
logFC_SLF_III_LIa:logFC_SLFIII_direc	−0.82	−3.84	2.22	70.17%	1.23%

## DISCUSSION

We recruited 153 left-handers to investigate the relationship between intrahemispheric white matter asymmetry, corpus callosum connectivity, and functional lateralization in language production and spatial attention. To assess fiber-specific measures, we performed an advanced fixel-based method and concluded three key findings. In terms of functional lateralization direction, language production exhibited group differences in SLF-III FDC asymmetry, whereas spatial attention displayed group differences in FDC of the rostrum and rostral body of the corpus callosum, suggesting that the lateralization direction of the two functions relies differently on inter- and intrahemispheric connectivity. However, no intrahemispheric tract asymmetry direction mirrored the direction of functional lateralization. In terms of functional lateralization degree, language production was mainly predicted by SLF-III FDC asymmetry and FDC of the rostral body of the corpus callosum, whereas spatial attention was mainly predicted by FC of the rostrum and FD of the anterior midbody of the corpus callosum, indicating that the degree of two functional lateralizations are affected differently by within and between hemispheric interaction. Ultimately, callosal connectivity had a negative effect on the degree of functional lateralization in both language production and spatial attention, which supports the hypothesis that the corpus callosum plays an excitatory role in functional lateralization.

### Behavioral Laterality Based on Visual Half Fields Naming Tasks and Brain Lateralization Based on fMRI Task

In line with previous study by Van der Haegen et al. (2011), we observed a high hit ratio in detecting individuals with atypical and typical language lateralization in VHF tasks. In addition, our results also showed a moderate false alarm rate, which reflects the divergence between brain lateralization and behavioral laterality. This was particularly observed in individuals with typical language lateralization who exhibited symmetrical behavioral laterality. This phenomenon can be attributed to two factors. First, the fMRI and behavioral task used different paradigms (verbal fluency vs. word/picture naming). The fMRI task measured core language-related components by contrasting with a baseline condition that removed basic sensory and motor processes. In contrast, the behavioral task required additional cognitive processes, such as word recognition, which may not consistently align with the hemisphere specialized for language production in all participants (Van der Haegen et al., 2012). This could potentially result in reduced behavioral laterality. Second, the behavioral task required overt language production, while the fMRI task required covert production, which could also contribute to the variations in lateralization measures. Despite the variation observed between behavioral and brain laterality, the behavioral tasks were beneficial for enhancing the effectiveness and economic efficiency of identifying target individuals compared to direct resource-intensive fMRI methods.

### Lateralization Direction and Intra- and Interhemispheric White Matter Connectivity in Language and Visual Spatial Attention

Our analysis revealed no significant differences in the asymmetry of the arcuate fasciculus between the two language dominance groups, with respect to both direction and degree. This finding is in concordance with previous studies investigating left-handers. Specifically, Vernooij et al. (2007) demonstrated a consistent lateralization direction of arcuate fasciculus fiber density across both atypical and typical language lateralization groups. Likewise, Verhelst et al. (2021) and Gerrits et al. (2022) reported no significant differences in the laterality of arcuate fasciculus FDC laterality. These observations suggest that arcuate fasciculus asymmetry may not be a crucial factor in determining language lateralization. An interesting finding was related to the asymmetry of the SLF-III. Although its asymmetry direction is independent of the direction of functional lateralization, our results revealed that individuals with RLD exhibited less leftward asymmetries in the SLF-III FDC compared to those with left language dominance. This observed difference in asymmetry can be attributed to stronger left SLF-III connectivity in individuals with typical lateralization. These findings suggest that it is the degree of asymmetry in the SLF-III, rather than its direction, that plays a critical role in language lateralization. The SLF-III, originating from the supramarginal gyrus and terminating in the inferior frontal cortex, plays a critical role in auditory–articulation mapping during phonological production and maintenance of verbal information (Barbeau et al., 2020; Nakajima et al., 2020; Shekari & Nozari, 2023). Evidence of the lateralization of SLF-III has been inconclusive from previous literature. Many previous studies used morphological measures such as volume to characterize the structural properties of SLF-III and observed a rightward asymmetry (Bakhit et al., 2022; Hecht et al., 2015; Howells et al., 2018; Thiebaut de Schotten et al., 2011), while others used DTI-based measures such as mean diffusion yet reported a leftward asymmetry (Budisavljevic et al., 2017; Makris et al., 2005). Based on the direction of the asymmetry of the fiber tract, its function was assumed to be related to language or spatial attention. Given the variability of tract measurements, it has been suggested that establishing a definitive link between specific tract asymmetry and functional lateralization based on collateralization at the population-level is insufficient (Vernooij et al., 2007). The current study overcame this limitation by directly examining the effect of lateralization direction and degree on tract asymmetry at the individual level. Our findings uncovered a significant difference in SLF-III asymmetry between two language dominance groups, although no such difference was found with respect to the lateralization of spatial attention. This finding suggests that the asymmetry characteristic of SLF-III is more likely related to the lateralization of language production than to spatial attention. This is further supported by a recent study that showed reduced functional connectivity between the left inferior frontal gyrus and other language regions (particularly the inferior parietal region) in individuals with RLD compared to those with LLD during the resting state (Wang et al., 2019). Notably, no significant difference was found between groups in any segment of the corpus callosum. This indicates that the direction of lateralization for language production relies more on the intrahemispheric white matter asymmetry than on interhemispheric white matter connectivity. This is further supported by the work of Keller and Kell (2016), who found an association between left lateralization and enhanced intrahemispheric coupling, as opposed to contralateral processing interactions.

Our study found that SLF-III and arcuate fasciculus have different roles in language lateralization, which can be attributed to two main factors. First, the anatomical connectivity differences between these tracts play a crucial role. SLF-III links the frontal regions with the supramarginal gyrus, which is important for articulatory processing and phonological encoding in language production. The arcuate fasciculus connects the frontal and temporal lobes, which facilitates the phonological, semantic, and syntactic processes (Yagmurlu et al., 2016). This difference underpins the varying roles of these tracts in language processing. Second, the specific nature of the language tasks used in our study may have differentially engaged these two tracts. The word production task (Pinyin vs. baseline contrast) activated the parietal-frontal network (Bradshaw et al., 2017), which is prominently connected by SLF-III.

For spatial attention, in line with the findings of previous studies (Labache et al., 2020; Villar-Rodríguez et al., 2024) that found a link between atypical functional lateralization and increased callosal volume, we revealed that significant group differences emerged in the FDC and logFC of two segments of the corpus callosum: CC-1 (rostrum) and CC-3 (rostral body). Individuals with atypical left dominance have a larger fiber cross-section compared to individuals with typical right dominance. CC-3 bridges the bilateral premotor area, which includes the frontal eye field (FEF), a region known to control saccadic eye movements and direct visual spatial attention (for review, see Fiebelkorn & Kastner, 2020). Early studies in monkeys showed that damage to the FEF led to deficits in gaze orientation to the contralesional hemifield and in attention ability (Welch & Stuteville, 1958). Electrophysiological studies further confirmed that the stimulation of FEF neurons evoked not only saccade production but also attention-related increases in neural activity in the visual cortex, even without saccades or post microsaccades (Armstrong et al., 2006; Liu et al., 2022; Moore & Armstrong, 2003; Schafer & Moore, 2011). In humans, the FEF has also been found to engage with the intraparietal gyrus/superior parietal lobule in goal-directed attention processing (Corbetta & Shulman, 2002, 2011). These findings indicate that covert spatial attention is closely linked to the oculomotor system. On the other hand, CC-1, which connects the bilateral orbitofrontal cortex, is involved in value-based decision making, emotional regulation, and inhibition control (Knudsen & Wallis, 2022; Rudebeck & Rich, 2018; Stalnaker et al., 2015; Wallis, 2007). Interestingly, these functions are similar to spatial attention and show a rightward bias at the population level (Aron et al., 2003; Gerrits, Verhelst, & Vingerhoets, 2020; Karolis et al., 2019). Thus, the orbitofrontal connection may not reflect the lateralization direction of spatial attention, but rather reflect collateral functions within the same hemisphere. Future research focusing on left-handers and combining task-based fMRI with diffusion analysis, is needed to provide novel insights into the individual-level interaction pattern of lateralized functions.

Our research shed light on a complementary pattern of the relationship between white matter connections and the directions of the two functional lateralizations. Specifically, the direction of language lateralization was associated with intrahemispheric fiber connections (SLF-III), whereas the direction of spatial attention lateralization was impacted by interhemispheric connections. This aligns with Semmes’s (1968) proposition that language and motor processing in the left hemisphere requires a more localized representation for intricate sensorimotor control, whereas spatial orientation in the right hemisphere needs a diffuse representation to foster multimodal coordination. This is further supported by a resting-state fMRI study showing that language and motor regions tend to interact within the hemispheres, while spatial and attentional regions tend to interact across hemispheres (Gotts et al., 2013). This different reliance of the lateralization direction of language and spatial attention on intra- and interhemispheric connectivity may represent an underlying anatomical mechanism responsible for the complementary pattern of language production and spatial attention lateralization, which was revealed by previous studies (Badzakova-Trajkov et al., 2010; Cai et al., 2013). These findings provide new insights into potential mechanisms underlying atypical functional lateralization in clinical populations, and pave the way for future research to explore the reciprocal effects of lateralized functions and the associated intra- and interhemispheric connectivity pattern.

### Degree of Functional Lateralization and Intra- and Interhemispheric White Matter Connectivity in Language and Spatial Attention

The degree of asymmetry in SLF-III FDC was linked not only to the degree but also to the direction of functional lateralization, suggesting a central role of SLF-III in language lateralization (see Lateralization Direction and Intra- and Interhemispheric White Matter Connectivity in Language and Visual Spatial Attention for details). Furthermore, the degree of lateralization was mainly predicted by the rostral body of the corpus callosum (CC-3) in the FD and FDC model. It is well established that the bilateral premotor areas connected by CC-3 are involved in motor planning and execution in language production (Hickok et al., 2011). The premotor areas has been found to play various roles in language processing, including behaviors such as action imitation in language perception (Liberman & Mattingly, 1985; Watkins et al., 2003), interpretation of gesture information in language contexts (Willems et al., 2007), and involvement in rhythm processing (Riecker et al., 2002). For spatial attention, the degree of lateralization was predicted by the anterior midbody of the corpus callosum (CC-4) in FD models and CC-1 (rostrum) in logFC model. CC-4 links the bilateral primary motor area. Although not the core region for spatial attention, the motor area may be involved in motor planning and making responses based on the task demands (Rushworth et al., 2001). And CC-1 was associated with both the direction and the degree of spatial attention lateralization (for details, see Lateralization Direction and Intra- and Interhemispheric White Matter Connectivity in Language and Visual Spatial Attention). Overall, the degree of language lateralization was predicted by both intra- (SLF-III) and interhemispheric white matter connections, while spatial attention was primarily predicted by interhemispheric connections.

Notably, our research showed a negative relationship between the degree of functional lateralization and the connection of rostral body and rostrum of corpus callosum in both language production and spatial attention. This finding provides more direct evidence than prior studies that relied on behavioral measures of brain lateralization and structural connections (Gootjes et al., 2006; Yazgan et al., 1995). In contrast to the positive association reported by Josse et al. (2008), which was based on the midsagittal area of the corpus callosum, our study used fixel-based metrics, which allows for greater fiber specificity. Our findings align with the hypothesis proposed by Ringo et al. (1994), suggesting that the increase in brain size during evolution necessitated the development of functional lateralization to minimize time delays in the interhemispheric transfer of information. Importantly, we provide supportive evidence for the excitatory hypothesis of functional lateralization of the corpus callosum. Stronger functional lateralization was found to correspond to weaker connectivity between bilateral regions across individuals. A similar pattern was also observed at the regional level within individuals, with increased lateralization of brain regions correlating with decreased callosal connections to regions on each side (Karolis et al., 2019).

Overall, our findings indicated that the direction of tract asymmetries did not mirror the direction of functional lateralization. Rather, the data revealed that the functional–structural relationship was more influenced by the degree of laterality and callosal connectivity. This suggests that functional lateralization may rely more on a complex interplay of intra- and interhemispheric structural networks, rather than mere tract asymmetries. This perspective is supported by several studies at the level of functional connectivity. For instance, Wang et al. (2019) identifies reduced left-lateralized connectivity within the language systems in individuals with atypical language dominance. In parallel, Labache et al. (2020) observed the engagement of both the left and right language networks, without mirrored pattern in the atypical lateralization group. This was accompanied by increased interhemispheric callosal connectivity. Moreover, in the realm of face processing, Wang et al. (2020) demonstrated that the degree of lateralization was closely linked with the ratio of intra- and interhemispheric structural connections. The dissociation of function and tracts symmetries for atypical individuals may reflect a flexible engagement of multiple structural pathways, potentially facilitated by increased callosal connection (Labache et al., 2020). Such a structure–function relationship likely underlies the mechanisms influencing the function-behavioral association, aligning with Rogers (2017), who emphasizing the evolutionary significance of lateralization degree over directional symmetry.

### Limitations

Given the rarity of atypical lateralization in right-handed individuals and its relatively higher occurrence in left-handed ones (Knecht et al., 2000), focusing on left-handers allows for the investigation of white matter connectivity in different lateralization directions. Nevertheless, the study exclusively involved left-handed participants, leaving uncertainty regarding whether handedness could potentially affect the relationship between functional lateralization and anatomical asymmetry (Bakhit et al., 2022). Further, functional lateralization was determined based on the core structural regions associated with language production and spatial attention. This captures the anatomical asymmetry tied to local properties, but overlooks the connectivity among brain regions. An alternative way to characterize functional lateralization is to investigate at the connectional level, which can reflect the network properties of isolated regions (Bartha-Doering et al., 2021; Stephan et al., 2007). For a more comprehensive understanding of the anatomical mechanism underlying functional lateralization, future research can combine the models of functional connectivity (e.g., psychophysiological interactions and dynamic causal modeling) with white matter connectivity (Joliot et al., 2016; Tzourio-Mazoyer & Seghier, 2016). In addition, it should be noted that the intrahemispheric tracts of interest TractSeg reconstructed introduced biases in our study (Vingerhoets et al., 2023). Although efforts to mitigate this through the adoption of a symmetrical FOD template, the potential for asymmetry bias remains. Future advancements in tractography, particularly in a fixel-based framework accommodating symmetrical tracts, are expected to address these concerns. Lastly, we employed a threshold of LI = 0 to define groups with left or right language lateralization. However, this threshold did not allow for the differentiation of a subgroup with bilateral language dominance (BLD). We also explored alternative thresholds (0.3, 0.4, and 0.5) to define BLD and strongly lateralized groups. Similar significant group effects on SLF-III asymmetry were observed, with the BLD group exhibiting less leftward asymmetry compared to the typical group, with no differences from the right lateralization group. Future research should further investigate the thresholds used in fMRI and their alignment with established clinical standards to enhance the clinical relevance of lateralization studies.

## CONCLUSION

The present study set out to investigate the relationship between white matter connections and functional lateralization in language production and spatial attention in left-handers. For the direction of lateralization, individuals with LLD and RLD differed in intrahemispheric asymmetry (i.e., SLF-III), but not in any part of the corpus callosum, whereas individuals with left and right spatial attention dominance differed in the rostrum and rostral body of the corpus callosum, but not in SLF-II/III asymmetry. This varying dependence of the two functions on intra- and interhemispheric connectivity may contribute to their complementary lateralization. Regarding the degree of lateralization, less leftward of SLF-III asymmetry is associated with more rightward language lateralization, suggesting the important role of the parietal-frontal connection in language lateralization. Moreover, the anterior parts of the corpus callosum connecting bilateral motor-related regions positively predicted the degree of lateralization in both functions, supporting the excitatory model of the corpus callosum in functional lateralization. This study extends our understanding of the relationship between function and anatomy from a lateralization perspective and may have some implications for clinical populations with atypical lateralization.

## ACKNOWLEDGMENTS

We are grateful to Prof. Chu-Chung Huang and Yanlin Yu for their suggestions on data processing.

## FUNDING INFORMATION

Qing Cai, National Natural Science Foundation of China (http://dx.doi.org/10.13039/501100001809), Award ID: 31970987. Qing Cai, National Natural Science Foundation of China (https://dx.doi.org/10.13039/501100001809), Award ID: 31771210.

## AUTHOR CONTRIBUTIONS

**Miaomiao Zhu**: Conceptualization: Equal; Data curation: Lead; Formal analysis: Lead; Investigation: Lead; Methodology: Lead; Project administration: Equal; Software: Lead; Validation: Lead; Visualization: Lead; Writing – original draft: Lead; Writing – review & editing: Lead. **Xiao Wang**: Investigation: Supporting; Methodology: Supporting. **Xier Zhao**: Writing – review & editing: Supporting. **Qing Cai**: Conceptualization: Equal; Funding acquisition: Lead; Project administration: Lead; Resources: Lead; Supervision: Lead; Writing – review & editing: Equal.

## DATA AND CODE AVAILABILITY STATEMENT

The processed task fMRI data and behavioral stimuli are available on OSF (https://osf.io/tpr4b/). T1 and diffusion data will be available upon reasonable request. Due to privacy concerns and ongoing changes in our institution’s ethical guidelines, T1 and diffusion data are currently not publicly available. The fMRI data preprocessing can be replicated using SPM12 (https://www.fil.ion.ucl.ac.uk/spm/). Head movements were evaluated using the ART toolbox (https://www.nitrc.org/projects/artifact_detect/). Lateralization index was calculated with the LI toolbox (https://www.medizin.uni-tuebingen.de/de/das-klinikum/einrichtungen/kliniken/kinderklinik/kinderheilkunde-iii/forschung-iii/software). Preprocessing of the diffusion data and the calculation of fixel-based metrics followed the MRtrix3 pipeline (https://mrtrix.readthedocs.io/en/latest/). Tractography was performed using TractSeg (https://github.com/MIC-DKFZ/TractSeg). Statistical analysis and visualization were performed using the R packages (Version 4.2.2; https://www.r-project.org/). Public codes were used for the imaging data analysis, and the sample code for behavioral data analysis is available on the OSF (https://osf.io/tpr4b/).

## Supplementary Material



## TECHNICAL TERMS

Functional lateralization: A bias for one side of the brain to dominate certain neutral functions or cognitive processes.
Gray matter asymmetry: Variations in gray matter volume, cortical thickness, and other morphological metrics between a left region and its right homologue.
Fixel: A specific fiber population within a voxel.
