# Supplementary material for: Intrahemispheric White Matter Asymmetries and Interhemispheric Connections Underlying the Lateralization of Language Production and Spatial Attention in Left-Handers
Source: Neurobiol Lang (Camb). 2025 Jan 10;6:nol_a_00153. doi: 10.1162/nol_a_00153 (PMC11740161; doi:10.1162/nol_a_00153)
Supplement: Supplementary file 1 [file nol-6-nol_a_00153-s001.pdf]

Supplementary Figure 1

The Relationship Between Handedness, Functional Lateralization in Language Production and Behavioral Laterality in Word and Picture Naming Tasks

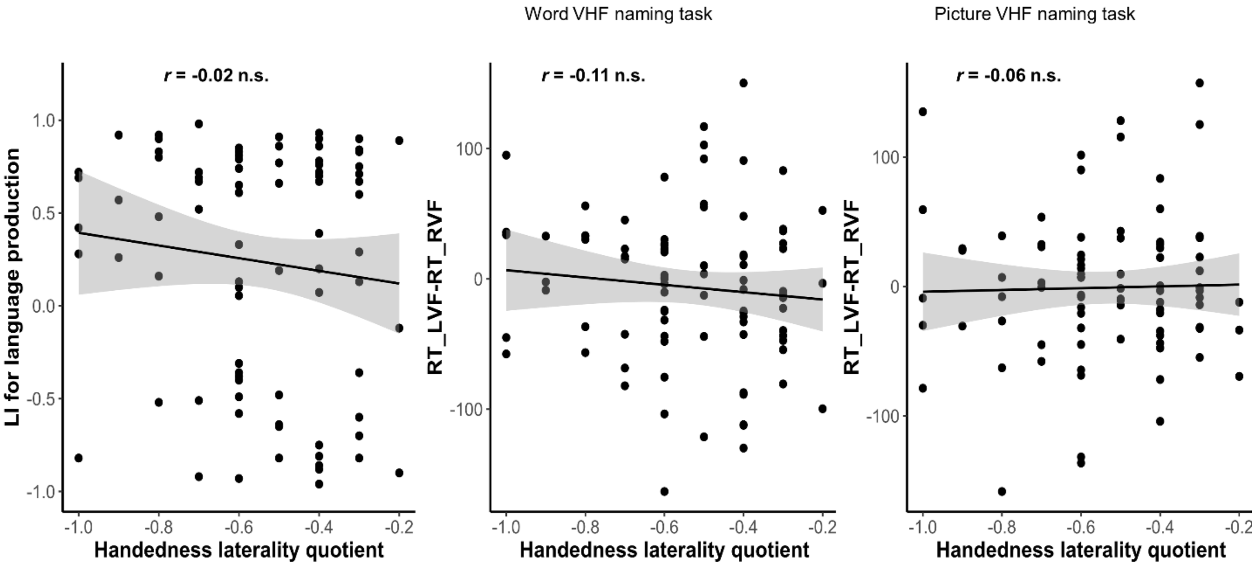

Note. The scatter plots illustrate the absence of significant correlations between the handedness laterality quotient and the lateralization index (LI) for language production based on Broca's area activation (Left). No significant correlations were found between the handedness laterality quotient and reaction time (RT) differences observed in the word (Middle) and picture (Right) naming tasks across visual half fields (VHF). The lateralization index (LI) quantifies the degree of functional lateralization in brain activation, while RT RT_LVF-RT_RVF reflects the reaction time differences between the left and right VHF in the naming tasks. n.s. denotes not significant statistics ($p > 0.05$).

Supplementary Table 1

Results of Spearman's Rank Correlations Between Intra-Hemispheric Tracts Asymmetry (FDC) and Functional Laterality for Spatial Attention

Variables	*r*	95% CI		*p*	*p*_adj	*p*_reg
		LL	*UL*			
FDC						
SLF_III_AI	-0.29	-0.47	-0.07	.008	.024	.378
SLF_II_AI	0.09	-0.13	0.3	.406	.609	.930
FD						
SLF_III_AI	-0.06	-0.28	0.16	.558	.670	.979
SLF_II_AI	-0.03	-0.25	0.19	.785	.785	.371
logFC						
SLF_III_AI	-0.33	-0.51	-0.12	.002	.012	.153
SLF_II_AI	0.11	-0.11	0.32	.324	.609	.140

Note. LI lateralization index, FDC fiber density and cross section, FD fiber density, FC fiber cross section, r Spearman correlation coefficient, CI confidence interval, SLF_III_AI superior longitudinal fasciculus III asymmetry index, SLF_II_AI superior longitudinal fasciculus II asymmetry index, p uncorrected p value, P adj Adjusted p-value using the Benjamini-Hochberg procedure for multiple testing. *P*_reg the p-value obtained after regressing out the lateralization index for language production.

Supplementary Table 2

Results of χ^2 Test for the Relation Between SLF_III Asymmetry Directions and Functional Lateralization Directions in Language Production

Measures	SLF_III_AI left	SLF_III_AI right	$\chi 2$	*p*	*p*_adj
FDC					
LI_left	51	9	2.53	.111	.222
LI_right	19	8			
FD					
LI_left	51	9	1.48	.224	.269
LI_right	20	7			
logFC					
LI_left	38	22	2.72	.099	.222
LI_right	12	15			

Note. FDC fiber density and cross section, FD fiber density, FC fiber cross section, LI_left: left lateralization dominance, LI_right right lateralization dominance, SLF_III_AI_left, left superior longitudinal fasciculus III asymmetry, SLF_III_AI_right, right superior longitudinal fasciculus III asymmetry, X^2 Chi-square test statistic, p uncorrected p value, *P*_adj Adjusted p-value using the Benjamini-Hochberg procedure for multiple testing.

Supplementary Table 3

Results of χ^2 Test for the Relation Between AF Asymmetry Directions and Functional Lateralization Directions in Language Production

Measures	AF_AI_left	AF_AI_right	$\chi 2$	*p*	*p*_adj
FDC					
LI_left	27	33	0.48	.487	.487
LI_right	10	17			
FD					
LI_left	21	39	5.92	.015	.090
LI_right	17	10			
logFC					
LI_left	34	26	1.89	.169	.254
LI_right	11	16			

Note. FDC fiber density and cross section, FD fiber density, FC fiber cross section, LI_left: left lateralization dominance, LI_right right lateralization, AF_AI_left, left arcuate fasciculus asymmetry, AF_AI_right, right arcuate fasciculus asymmetry direction, X^2 Chi-square test statistic, p uncorrected p value, *P*_adj Adjusted p-value using the Benjamini-Hochberg procedure for multiple testing.

Supplementary Table 4

Results of χ^2 Test for Relation Between SLF_II Asymmetry Directions and Functional Lateralization Directions in Spatial Attention

Measures	SLF-II-AI left	SLF-II-AI right	$\chi 2$	*p*	*p*_adj
FDC					
LI_left	24	11	3.89	.048	.144
LI_right	24	27			
FD					
LI_left	19	16	0.015	.902	.902
LI_right	27	24			
logFC					
LI_left	20	15	2.12	.145	.290
LI_right	21	30			

Note. FDC fiber density and cross section, FD fiber density, FC fiber cross section, LI_left: left lateralization dominance, LI_right right lateralization dominance, SLF_II_AI_left, left superior longitudinal fasciculus II asymmetry, SLF_II_AI_right, right superior longitudinal fasciculus II asymmetry, X^2 Chi-square test statistic, p uncorrected p value, *P*_adj Adjusted p-value using the Benjamini-Hochberg procedure for multiple testing.

Supplementary Table 5

Results of χ^2 Test for Relationship Between SLF_III Asymmetry Directions and Functional Lateralization Directions in Spatial Attention

Measures	SLF_III_AI left	SLF_III_AI right	$\chi 2$	*p*	*p*_adj
FDC					
LI_left	25	10	1.44	.230	.345
LI_right	42	9			
FD					
LI_left	29	6	0.08	.773	.902
LI_right	41	10			
logFC					
LI_left	11	24	6.24	.012	.072
LI_right	30	21			

Note. FDC fiber density and cross section, FD fiber density, FC fiber cross section, LI_left: left lateralization dominance, LI_right right lateralization dominance, SLF_III_AI_left, left superior longitudinal fasciculus III asymmetry, SLF_III_AI_right, right superior longitudinal fasciculus III asymmetry, X^2 Chi-square test statistic, p uncorrected p value, *P*_adj Adjusted p-value using the Benjamini-Hochberg procedure for multiple testing.

Supplementary Table 6

The Mean and SD of Metrics for SLF_III and AF in the Two Hemispheres for Two Spatial Attention Lateralization Groups

Measures	LLD (*N*=35)		RLD (*N*=51)		*W*	*p*	*p*_adj
	M	*SD*	*M*	*SD*			
FDC							
SLF_III_AI	-0.004	0.02	0.003	0.03	680	.089	.267
SLF_II_AI	0.002	0.03	-0.001	0.03	895	.259	.389
FD							
SLF_III_AI	0.001	0.02	-0.001	0.02	847	.583	.664
SLF_II_AI	-0.002	0.02	0.001	0.02	780	.664	.664
logFC							
SLF_III_AI	-0.011	0.03	0.007	0.04	624	.031	.186
SLF_II_AI	0.007	0.05	-0.005	0.05	917	.197	.389

Note. FDC fiber density and cross section, FD fiber density, FC fiber cross section, SLF_III_AI superior longitudinal fasciculus III asymmetry index, SLF_II_AI superior longitudinal fasciculus II asymmetry index, RSD right spatial attention dominant group, LSD left spatial attention dominant group, W Mann–Whitney statistic, p: uncorrected p value, p adj: Adjusted p-value using the Benjamini-Hochberg procedure for multiple testing.

Supplementary Table 7

Results of the Group Comparisons of the Connectivity of the Corpus Callosum Subdivisions for Language Production

Measures	LLD (*N*=60)		RLD (*N*=27)		*W*	*p*
	M	*SD*	*M*	*SD*		
FDC						
CC_1	0.42	0.04	0.43	0.05	677	.890
CC_2	0.41	0.03	0.42	0.04	744	.729
CC_3	0.45	0.04	0.46	0.06	754	.698
CC_4	0.50	0.04	0.50	0.03	805	.520
CC_5	0.47	0.04	0.46	0.03	878	.268
CC_6	0.45	0.04	0.44	0.04	948	.104
CC_7	0.46	0.05	0.45	0.05	925	.147
FD						
CC_1	0.40	0.02	0.40	0.02	710	.822
CC_2	0.40	0.01	0.39	0.01	862	.318
CC_3	0.44	0.02	0.43	0.02	970	.072
CC_4	0.48	0.02	0.48	0.01	806	.516
CC_5	0.46	0.02	0.46	0.01	876	.272
CC_6	0.42	0.02	0.42	0.02	859	.328
CC_7	0.41	0.02	0.41	0.02	822	.458
logFC						
CC_1	0.04	0.07	0.06	0.09	717	.805
CC_2	0.05	0.06	0.06	0.08	742	.735
CC_3	0.03	0.07	0.06	0.10	684	.877
CC_4	0.03	0.06	0.03	0.06	784	.596
CC_5	0.02	0.06	0.01	0.06	878	.268
CC_6	0.06	0.06	0.04	0.08	967	.076
CC_7	0.13	0.07	0.11	0.09	948	.104

Note. FDC fiber density and cross section, FD fiber density, FC fiber cross section, CC_1 to CC_7 subdivisions of the corpus callosum, RLD right language dominant group, LLD left language dominant group, W Mann–Whitney statistic, p: uncorrected p value, p adj: Adjusted p-value using the Benjamini-Hochberg procedure for multiple testing.

Supplementary Table 8

Results of Bayesian Regression Analysis for White Matter Variables (LogFC) Predicting the Degree of Functional Lateralization for Language Production

Parameters	*Median*	95% CI		*pd*	*% in ROPE*
		LL	*UL*		
(Intercept)	0.85	0.35	1.34	99.95%	*<0.01%*
gender	-0.05	-0.18	0.08	78.11%	23.17%
age	-0.01	-0.03	0.01	78.99%	95.54%
logFC_AF_LIa	-3.08	-7.24	1.05	92.95%	0.34%
logFC_SLF_III_LIa	-0.97	-4.26	2.32	72.10%	1.04%
logFC_CC1	-0.53	-1.88	0.83	78.05%	2.27%
logFC_CC2	0.22	-1.92	2.35	58.08%	1.88%
logFC_CC3	0.29	-1.07	1.64	66.35%	2.89%
logFC_CC4	1.11	-0.92	3.16	85.98%	1.12%
logFC_CC5	-1.45	-3.68	0.76	90.33%	0.77%
logFC_CC6	1.06	-0.63	2.76	89.35%	1.13%
logFC_CC7	-0.28	-1.41	0.85	68.84%	3.28%
logFC_AF_LIa:logFC_AFLI_direc	1.29	-3.3	5.9	71.00%	0.74%
logFC_SLF_III_LIa:logFC_SLFIII_direc	2.86	-0.44	6.17	94.52%	0.28%

Note. Median estimate of posterior distributions, 95% CI 95% credibility intervals, pd, probability of direction quantifies the effect's directional likelihood, correlating with p-values (pd of 95% ≈ $p < .1$; 97.5% ≈ $p < .05$; 99.5% ≈ $p < .01$; 99.95% ≈ $p < .001$), % in ROPE region of practical equivalence with <1% considered significant for rejecting the null hypothesis. logFC_AF_LIa: the absolute of AF logFC asymmetry. logFC_SLF_III_LIa: the absolute of SLF_III logFC asymmetry. logFC_AF_LIa:logFC_AFLI_direc: the interaction term between the language function-AF asymmetry directionality consistency and the AF asymmetry degree. logFC_SLF_III_LIa:logFC_SLFIII_direc: the interaction term between the language function-SLF-III asymmetry directionality consistency and the SLF-III asymmetry

degree.

Supplementary Table 9

Results of Bayesian Regression Analysis for White Matter Variables (FDC) Predicting the Degree of Functional Lateralization for Spatial Attention

Parameters	*Median*	95% CI		*pd*	*% in ROPE*
		LL	*UL*		
(Intercept)	1.56	0.5	2.61	99.75%	<0.01%
gender	<-0.01	-0.13	0.13	50.57%	31.96%
age	<0.01	-0.02	0.03	63.02%	99.73%
FDC_SLF_II_LIa	-0.34	-3.63	2.97	58.16%	1.29%
FDC_CC1	-1.46	-3.84	0.92	88.98%	0.83%
FDC_CC2	1.68	-2.93	6.27	76.67%	0.70%
FDC_CC3	0.31	-2.18	2.79	59.76%	1.65%
FDC_CC4	-1.41	-4.6	1.76	81.12%	0.91%
FDC_CC5	-0.93	-4.69	2.8	68.91%	0.99%
FDC_CC6	-1.13	-4.46	2.19	75.02%	1.01%
FDC_CC7	1	-1.21	3.22	81.51%	1.27%
FDC_SLF_III_LIa	-0.46	-4.4	3.48	59.23%	1.01%
FDC_SLF_II_LIa:FDC_SLF_IILI_direc	-0.76	-4.09	2.57	67.42%	1.15%
FDC_SLF_III_LI:FDC_SLFIII_direc	-2.21	-5.38	0.96	91.56%	0.49%

Note. Median estimate of posterior distributions, 95% CI 95% credibility intervals, pd, probability of direction quantifies the effect's directional likelihood, correlating with p-values (pd of 95% ≈ p < .1; 97.5% ≈ p < .05; 99.5% ≈ p < .01; 99.95% ≈ p < .001), % in ROPE region of practical equivalence with <1% considered significant for rejecting the null hypothesis. FDC_SLF_II_LIa: the absolute of SLF_II FDC asymmetry. FDC_SLF_III_LIa: the absolute of SLF_III FDC asymmetry. FDC_SLF_II_LIa:FDC_SLFIILI_direc: the interaction term between the language function-SLF_II asymmetry directionality consistency and the SLF_II asymmetry degree. FDC_SLF_III_LIa:FDC_SLFIII_direc: the interaction term between the language function-SLF-III asymmetry directionality consistency and the SLF-III

asymmetry degree.

Supplementary Table 10

The Mean and SD of Metrics for SLF_III and AF in the Two Hemispheres for Two Language Lateralization Groups

Measures	LLD (*N*=60)				RLD (*N*=27)			
	L-hemisphere		R-hemisphere		L-hemisphere		R-hemisphere	
	M	*SD*	*M*	*SD*	*M*	*SD*	*M*	*SD*
FDC								
SLF_III	0.39	0.04	0.37	0.03	0.38	0.04	0.37	0.04
AF	0.38	0.03	0.38	0.03	0.38	0.04	0.38	0.04
FD								
SLF_III	0.36	0.02	0.34	0.01	0.35	0.02	0.34	0.02
AF	0.35	0.01	0.35	0.01	0.35	0.01	0.35	0.02
logFC								
SLF_III	0.09	0.07	0.07	0.06	0.07	0.08	0.07	0.10
AF	0.08	0.05	0.08	0.06	0.07	0.08	0.08	0.09

Note. FDC fiber density and cross section, FD fiber density, FC fiber cross section, r Spearman correlation coefficient, SLF_III superior longitudinal fasciculus III, AF arcuate fasciculus, RLD right language dominant group, LLD left language dominant group.

A List of abbreviations for key terminologies

- **SLF-III**: Superior Longitudinal Fasciculus-III
- **AF**: Arcuate Fasciculus
- **GMA**: Gray Matter Asymmetry
- **PT**: Planum Temporale
- **IFG**: Inferior Frontal Gyrus
- **DTI**: Diffusion Tensor Imaging
- **CC**: Corpus Callosum
- **FBA**: Fixel-Based Analysis
- **CSD**: Constrained Spherical Deconvolution
- **FOD**: Fiber Orientation Distribution
- **FD**: Fiber Density
- **FC**: Fibre-Bundle Cross-Section
- **FDC**: Fiber density and cross-section area
- **VHF**: Visual Half-Field
- **VFA**: Visual Field Advantage (calculated by reaction time differences between the left and right visual field)
- **LVF**: Left Visual Field
- **RVF**: Right Visual Field
- **LI**: Lateralization Index (below 0 indicates right functional lateralization dominance, above 0 indicates left functional lateralization dominance; the closer to -1 or 1, the stronger the dominance)

- **LLD**: Left Language Dominance
- **RLD**: Right Language Dominance
- **LSD**: Left Spatial Attention Dominance
- **RSD**: Right Spatial Attention Dominance
- **TOI**: Tracts of Interest
- **AI**: Asymmetry Index (assessed by (left – right) / (left + right); a positive asymmetry index indicates leftward asymmetry in the white matter tract, whereas a negative asymmetry index indicates rightward asymmetry)
